# Modeling ALS in a dish: how organoids are transforming research

**DOI:** 10.3389/fmed.2026.1792336

**Published:** 2026-04-07

**Authors:** Gaia Galluzzi, Giancarlo Ruocco, Ersilia Fornetti, Ilaria Genovese

**Affiliations:** 1 Center for Life Nano and Neuro Science, Istituto Italiano di Tecnologia, Rome, Italy; 2 School of Advanced Studies, University of Camerino, Camerino, Italy; 3 Department of Physics, Sapienza University of Rome, Rome, Italy; 4 Departmental Faculty of Medicine, UniCamillus-Saint Camillus International University of Health Sciences, Rome, Italy

**Keywords:** ALS, disease modeling, neuromuscular organoids, spinal organoids, tridimensional cell cultures

## Abstract

Amyotrophic Lateral Sclerosis (ALS) is a rapidly progressive neurodegenerative disease characterized by the selective loss of upper and lower motor neurons, leading to muscle weakness, paralysis, and ultimately respiratory failure. The multifactorial etiology of ALS, encompassing genetic mutations, protein aggregation, oxidative stress, excitotoxicity, and dysregulated RNA metabolism, has hindered the development of effective therapies. Traditional animal and 2D cell models have provided important mechanistic insights but often fail to fully capture the human-specific and multicellular aspects of disease pathophysiology. Recent advances in induced pluripotent stem cell (iPSC)-derived organoids offer a promising human-based platform for ALS research, enabling the generation of disease-relevant neural and neuromuscular subtypes in three-dimensional architectures. These models recapitulate key pathological features, including protein mis-localization, neuromuscular junction defects, synaptic impairments, and glial contributions to motor neuron degeneration, while also serving as platforms for drug screening and mechanistic studies. Importantly, spinal and neuromuscular organoids bridge the gap between simplified *in vitro* systems and the complex human nervous system, providing a unique framework to study ALS pathogenesis. This review provides a comprehensive overview of the various differentiation protocols, experimental strategies and key results obtained to date, with a primary focus on validating and benchmarking organoid models, while also highlighting their limitations, emerging clinical applications, translational potential, and opportunities for personalized therapeutic discovery.

## Introduction

Amyotrophic Lateral Sclerosis (ALS) is a devastating progressive neurodegenerative disease, which selectively affects both upper and lower motor neurons, resulting in neuromuscular junction (NMJ) degeneration, progressive muscle weakness and culminating in respiratory failure (1). Compared with other neurodegenerative diseases, ALS progresses more rapidly, with a life expectancy of 2–5 years from symptom onset (2). However, survival can vary significantly, with factors like age, site of symptom onset, and the presence of specific genetic mutations influencing prognosis (3). The diagnosis of ALS is mainly based on clinical presentation and electrophysiological criteria, with patients exhibiting a range of symptoms including progressive weakness, muscle atrophy, spasticity, dysarthria, dysphagia, and respiratory compromise (4). Emerging approaches such as fluid biomarkers, advanced neuroimaging, and genetic testing show promise in supporting earlier and more accurate diagnosis, although they are not yet fully validated or routinely implemented in clinical practice (5).

While a minority of ALS cases have hereditary origins, over 90% occur without a known family history and a clear genetic cause. Cases belonging to the first category are generally classified as familial ALS (fALS), while the others are classified as sporadic ALS (sALS) (6). Nevertheless, both types can involve genetic mutations, with some sporadic cases harboring genes also found in familial forms (7). For this reason, this simple classification is useful but can be misleading, as the distinction is often blurry. Importantly, regardless of whether ALS arises sporadically or in familial forms, genetic studies have revealed a range of disrupted cellular processes in motor neurons that are thought to contribute to disease pathogenesis. These include excitotoxicity, oxidative stress, protein aggregation, impaired axonal transport, DNA damage, mitochondrial dysfunction, and dysregulation of RNA metabolism (8). Together, these alterations highlight the multifactorial nature of ALS and suggest that its progression results from the interplay of several converging mechanisms.

Although our knowledge regarding ALS has dramatically progressed since the first descriptions of the disease in the mid-1800s, the outlining of a univocal diagnostic definition, the identification of reliable biomarkers and the in-depth understanding of the diverse genetic and pathological mechanisms still represent a serious challenge today (9). These difficulties mainly stem from the complexity and heterogeneity of the disease and unfortunately hinder the development of effective therapies.

Given the etiological complexity and phenotypic variability inherent in ALS, studying the disease mechanisms and developing effective therapies requires robust and physiologically relevant models that can recapitulate the key features of the human condition. In this review, after outlining the genes causative of the disease and briefly summarizing the main *in vivo* and *in vitro* models used in ALS research, we will focus on human induced pluripotent stem cells (hiPSCs)-derived neural organoids. They represent the most recent human-based systems for ALS modeling and hold great promise for dissecting disease mechanisms and advancing therapeutic discovery.

## The genetic landscape of ALS

The genetic architecture of ALS is highly complex, with more than 30 genes identified being implicated in disease pathogenesis. The incomplete penetrance and variable expressivity of these mutations, together with the influence of environmental factors, contribute to the remarkable clinical heterogeneity observed among patients (10).

Among the genes most frequently associated with fALS, mutations in *SOD1* were the first to be identified and remain a cornerstone for both clinical studies and experimental models (11). Mutations occurring in *SOD1* gene are usually single aminoacidic substitutions that affect the native folding of the protein leading to aggregation, gain of toxic functions and loss of the physiological function of the protein since it is sequestered in these cytoplasmic aggregates (12). The same process happens in other genes interested by substitutions like *TARDBP* and *FUS* which encode RNA binding proteins (13).

To date, the most common genetic abnormality in both familiar and sporadic forms of ALS regard *c9orf72* gene and particularly, the expansion of the GGGGCC hexanucleotide (HRE) in the first intron/promoter region (14). The repeat expansion reduces *c9orf72* transcription levels causing a loss of function of the protein and accumulation of dipeptide repeat proteins (DPRs) and RNA foci (15).

Importantly, mis-localization and aggregation of the *TARDBP*-encoded TDP-43 protein are observed in the majority of ALS cases, including those without *TARDBP* mutations, suggesting a central role for TDP-43 dysfunction in the pathogenesis of both familial and sporadic forms of the disease (16).

Beyond these major genes, several others—such as *OPTN, TBK1, VCP, and NEK1*—have been implicated in a subset of patients. These genes converge on diverse cellular pathways, including autophagy, vesicle trafficking, and cytoskeletal dynamics (17). Collectively, they reinforce the concept that ALS is not driven by a single molecular defect but rather by the disruption of multiple, interconnected processes.

Although these mutations account for only a subset of ALS cases, they have been instrumental in shaping the field of ALS research. They have provided essential tools for developing experimental models that recapitulate key pathological features of the disease, thereby offering valuable platforms for studying disease mechanisms and testing therapeutic strategies (18).

## Modeling ALS: from animals to human-based systems

As previously mentioned, the intricate interplay between genetic predisposition, environmental influences, and stochastic events underscores the multifaceted nature of ALS, rendering it a formidable challenge to model and treat effectively (10). In this section, we will provide a brief overview of the main experimental systems used to investigate ALS, ranging from animal models, which have long served as the foundation for preclinical studies, to human-derived *in vitro* systems, highlighting recent advances in hiPSC-based approaches.

### Animal models of ALS: insights and translational gaps

To unravel the pathogenic mechanisms of ALS, over the past two decades a wide range of animal models, including rodents, invertebrates, zebrafish, and primates, have been developed. The first and most widely used models were generated in rodents, particularly transgenic mice expressing mutant *SOD1* (G93A, G37R, G85R, and H46R) which became a cornerstone for the field (19–21). These systems provided crucial insights into oxidative stress, protein misfolding, excitotoxicity, axonal transport defects, and the non–cell-autonomous contributions of glia, and later expanded to include models based on *TARDBP, FUS*, and *C9orf72* mutations (22).

Subsequently, invertebrate models such as Drosophila and C. elegans were introduced, taking advantage of their genetic tractability, rapid life cycles, and suitability for large-scale modifier screens. These organisms have been particularly useful for identifying pathways involved in protein toxicity and neuronal stress (23). Around the same time, zebrafish emerged as an attractive vertebrate model: their transparent embryos and well-characterized neuromuscular system allowed detailed investigations of axonal development, motor neuron degeneration, and early pathogenic processes, as well as medium-throughput drug testing (24).

More recently, large animal models, including pigs and non-human primates, have been generated in an effort to better approximate the anatomical and physiological complexity of the human nervous system (25–27). While these systems hold promises for bridging the translational gap, their use remains limited due to technical, ethical, and economic challenges.

Overall, these models have been instrumental in dissecting specific aspects of ALS pathophysiology, such as motor neuron degeneration, protein aggregation, NMJ dysfunction, and glial involvement (28). They have also served as valuable platforms for preclinical drug screening, providing mechanistic insights and candidate interventions (29). However, despite these contributions, all current animal models possess intrinsic limitations. Most replicate only a portion of the ALS phenotype tied to specific genetic mutations, and none fully capture the complexity of sporadic or multifactorial forms of the disease. As a result, therapies that show promise in animal models have repeatedly failed to demonstrate efficacy in human patients (30, 31). This translational gap underscores the challenge of predicting clinical outcomes in ALS and highlights the need for human-relevant models capable of better recapitulating the full spectrum of pathological features observed in patients.

### Human-derived *in vitro* models of ALS

In order to eliminate the issue of species variability in the development of new therapeutics, animal models have been complemented by *in vitro* systems based on human cells. These approaches offer a valuable intermediate step between animal models and more advanced stem cell–derived platforms, as they allow the study of human-specific cellular processes in relatively simple and accessible systems. While they lack the ability to fully reproduce the complexity of the human nervous system, they have nevertheless provided important insights into ALS-related mechanisms and remain useful tools for hypothesis-driven investigations.

Several human-derived *in vitro* systems have been employed to investigate ALS-related mechanisms. Immortalized cell lines, such as neuroblastoma SH-SY5Y or HEK293 cells, have provided accessible and versatile platforms as they are easy to grow and genetically manipulate. They have been (and are) widely used for targeted mechanistic studies, such as the localization of mutated proteins (*SOD1, TDP-43, FUS*), analysis of oxidative stress pathways, autophagy, or RNA metabolism (32–34). Patient-derived fibroblasts and lymphoblasts have also been used as an easily accessible model to explore alterations in transcriptional profile, mitochondrial morphology and dynamics, metabolism, and mutated proteins localization (35, 36). Moreover, primary neural and glial cells obtained from post-mortem tissue have provided interesting data on neuronal vulnerability and interaction with glia, but are limited by the scarcity of material, sample heterogeneity, and the impossibility of long-term expansion (37, 38).

Altogether, these human-derived systems have proven valuable for dissecting selected disease mechanisms, but their inherent limitations in terms of cellular diversity, physiological relevance, and long-term maintenance restrict their translational potential (39). These shortcomings have stimulated the development of more advanced human-based models, most notably induced pluripotent stem cells (iPSCs), which now represent the most promising tools for bridging the gap between simplified cellular assays and the multifactorial nature of ALS in patients.

### iPSC-derived *in vitro* models of ALS

Since the landmark work of Takahashi and colleagues on somatic cells reprogramming in 2007 (40), iPSCs biology has progressed rapidly, bringing to the development of more efficient and refined reprogramming strategies (41). These advances have not only expanded the applications of hiPSCs across multiple disciplines, but have also transformed the landscape of ALS research, providing unprecedented opportunities to generate patient-specific neuronal populations *in vitro*. Unlike immortalized or primary human cells, hiPSCs offer a virtually unlimited and renewable source of disease-relevant cell types, such as motor neurons, astrocytes and myoblast (42–44). These hiPSC-derived cell types can be obtained using either transgene-free differentiation protocols, which follow developmental pathways but require longer times, or accelerated methods involving genetic manipulation, which provide faster access to target populations but sometimes at the cost of reduced physiological fidelity (42, 45, 46).

Importantly, a critical step in ALS modeling is the assessment of whether hiPSC-derived cells recapitulate key pathological features observed in patients. In particular, numerous phenotypic alterations have been documented in iPSC-derived motor neurons carrying ALS-causing mutations. These include reduced viability, neurite abnormalities, protein aggregation, mitochondrial dysfunction, and electrophysiological impairments (47). Moreover, co-culture systems combining iPSC-derived neurons with astrocytes or microglia have further demonstrated the non–cell autonomous nature of ALS, showing that glial cells can actively contribute to motor neuron degeneration (48).

Beyond these studies, hiPSC-derived motor neurons provide a valuable platform for drug discovery and screening, enabling the evaluation of candidate compounds in a human genetic context (49). Notably, some studies have reported partial rescue of disease phenotypes, such as reduced oxidative stress or improved axonal transport, upon pharmacological treatment, reinforcing the translational potential of these models (50, 51).

Despite these advances, several limitations constrain the impact of iPSC models, including difficult culture conditions and the need for specialized expertise, making them a much more challenging model than traditional systems (52). Another major challenge is the observed variability between lines, which can result from differences in genetic background, reprogramming methods, and differentiation protocols (53). Such variability often leads to heterogeneous or subtle phenotypes, making it difficult to draw consistent conclusions. To mitigate these issues, different approaches have been adopted. A rigorous way to control for inter-individual variability is the use of isogenic lines, created by introducing or correcting disease-causing mutations in the same genetic background (54, 55). Additionally, exposing iPSC-derived cultures to toxic stimuli, including glutamate, oxidative stressors, or mitochondrial poisons, has been employed to exacerbate latent disease phenotypes, thereby standardizing experimental conditions and enhancing the robustness of ALS-related readouts (56, 57).

Overall, while iPSC-derived models cannot fully recapitulate the complex cellular interactions and multicellular architecture of the human nervous system yet, they represent a powerful and versatile tool for dissecting ALS mechanisms and for bridging the gap between simplified *in vitro* assays and more physiologically relevant human-based platforms.

## Organoids as next generation models in ALS research

Neural organoids are self-organized three-dimensional neural cultures obtained from either embryonic stem cells or iPSCs. Since the first cerebral organoid study was published by Lancaster and collaborators in 2013 in which the 3D structure developed a variety of regional identities organized as discrete domains capable of influencing one another (58), researchers focused on obtaining region specific organoids such as midbrain, retina and hippocampus to unravel the physiology and the pathological processes associated within different parts of the CNS (59, 60). Organoids systems have already been successfully used to model a spectrum of motor neuron diseases beyond ALS, including spinal muscular atrophy (SMA), hereditary spastic paraplegia (HSP), and distal hereditary motor neuropathies (dHMN). In SMA organoids derived from patient iPSCs, pathological features such as impaired motor neuron differentiation, reduced survival and neuromuscular junction (NMJ) dysfunction have been recapitulated, together with pharmaceutics testing with demonstrable phenotypic rescue (61). Hereditary spastic paraplegia models using corticospinal neuron-enriched organoids reveal axonal trafficking defects and cytoskeletal abnormalities typical of SPG4 mutations, providing insights into upper motor neuron vulnerability (62). Neuromuscular organoids modeling dHMN and Charcot–Marie–Tooth disease show impaired NMJ formation, synaptic instability, and Schwann cell dysfunction, highlighting the importance of glia–neuron interactions in peripheral motor neuropathies (63). In this section, we will discuss the application of organoid technology to ALS research. We will give an introduction of spinal cord generation during the development *in vivo*, highlighting two main theories by which this process happens. We will then provide an overview of the different protocols to generate spinal organoids derived from hiPSCs and then we will focus on studies that have applied such organoids as experimental models for ALS, illustrating the novel insights they have contributed to our understanding of disease mechanisms.

### Spinal cord development during embryogenesis

*In vivo* development of the spinal cord happens during gastrulation, where the neuroectoderm becomes the neural plate and invaginates to form the neural tube. This process, called neurulation, is highly complex and depends on the spatiotemporal gradient-dependent regulatory effects of morphogens, which induce distinct and spatially defined gene expression patterns that lead to cell fate specification (64). The posterior neural plate developmental process and the differentiation of the spinal cord is nowadays debated. According to the classical viewpoint, called the activation-transformation model, all neural tissue is first induced as anterior (forebrain) and subsequently transformed to more posterior fates such as hindbrain and spinal cord (65). However, there is a more recent theory for which spinal cord identity is determined by a distinct population of cells, the neuromesodermic progenitors (NMPs) that co-express biomarkers of the early mesoderm and neural progenitors (66).

During the establishment of the anteroposterior (AP) and dorsoventral (DV) axes of the neural tube, cross-talk among major morphogenetic pathways—such as retinoic acid (RA), Fibroblast Growth Factors (FGFs), Wnt signals, Sonic Hedgehog (Shh) secreted from the notochord and the floor plate, and Bone Morphogenetic Proteins (BMPs) from the roof plate—integrates with the graded activation of *HOX* genes to specify neural identity. Along the DV axis, the opposing gradients of these molecules generate eleven discrete progenitor domains, each characterized by a unique transcription factor code that defines their competence and response to morphogens (67).

### Generating spinal cord organoids: protocols and design principles

Given this highly orchestrated process, the protocols to generate spinal cord organoids are very heterogeneous, but they typically involve three steps: embryoid bodies (EBs) formation, neural induction and neural maturation.

HiPSCs are cultivated and expanded as a monolayer under naive conditions to maintain their undifferentiated state. The generation of organoids relies on hiPSC **“**self-aggregation**”** capability to generate 3D structures that possess characteristics of the three germ layers ectoderm, endoderm and mesoderm, named **embryoid bodies** (68). To trigger this process, hiPSCs must undergo mechanical detachment and enzymatic dissociation. For spinal cord organoids formation, there are two ways to obtain EBs: the detachment of entire 2D hiPSCs colonies that enables the spontaneous formation of floating 3D spheres (69) while the alternative is single cell dissociation and forced tridimensional aggregation in low attachment plates (70).

The **induction** step consists in committing cells toward the desired fate. Although its central role, fate determination can be achieved before or after EBs formation based on the neurodevelopmental theory on which the protocol is based. To generate NMPs, the induction has to begin before EBs formation, hence, hiPSCs are exposed to a specific cocktail of factors [Wnt activators and Fibroblast Growth Factor (FGF)] which create a stable and caudalized neuromesodermal commitment and ihibit endodermal patterning. HiPSCs are treated for few days before the detachment of the colonies and the subsequent formation of EBs, thus giving a cell population already committed to a NMP fate that will generate neuromuscular organoids (NMOs) (65).

In other protocols to derive Brain or Spinal Organoids (SpO) that follow the activation-transformation theory, EBs undergo **Dual SMAD inhibition** using antagonists of TGF-β and BMP pathways (SB431542, Noggin or LDN193189). By blocking these pathways, hiPSCs exit the pluripotent state and default to a neuroectodermal lineage (71). Subsequently, Retinoic Acid first and Sonic Hedgehog (or functional analogs like Puromorphamine) later promote **caudalization** and **ventralization** generating organoids enriched in spinal cord cell populations (72, 73). Another methodological distinction concerns the use of extracellular matrix embedding free-floating aggregates favor axial elongation and global tissue expansion (69) whereas Matrigel embedding can stabilize apical–basal polarity and promote neurulation-like folding or lumen formation (72).

The **maturation phase** varies considerably among protocols both in duration and in the conditions required. Typically, induced organoids–whether embedded in an extracellular matrix or kept free-floating–are maintained in an environment that helps their growth in 3D and supplemented with several **neurotrophic and pro-survival factors**, as Brain Derived Neurotrophic Factor (BDNF) and Glial-cell derived Neurotrophic Factor (GDNF) to promote neuronal maturation, synaptogenesis and long-term viability (73). Reported maturation windows range from approximately 35 days to up to 9 months. However, prolonged culture does not necessarily correlate with higher specialization, as organoids eventually develop a necrotic core due to the absence of vascularization and the inadequate distribution of oxygen and nutrients (74). This necrosis can be delayed by using dynamic culture systems that enhance perfusion and nutrient exchange, such as spinner flasks or orbital shakers (58, 75).

**Figure 1:**
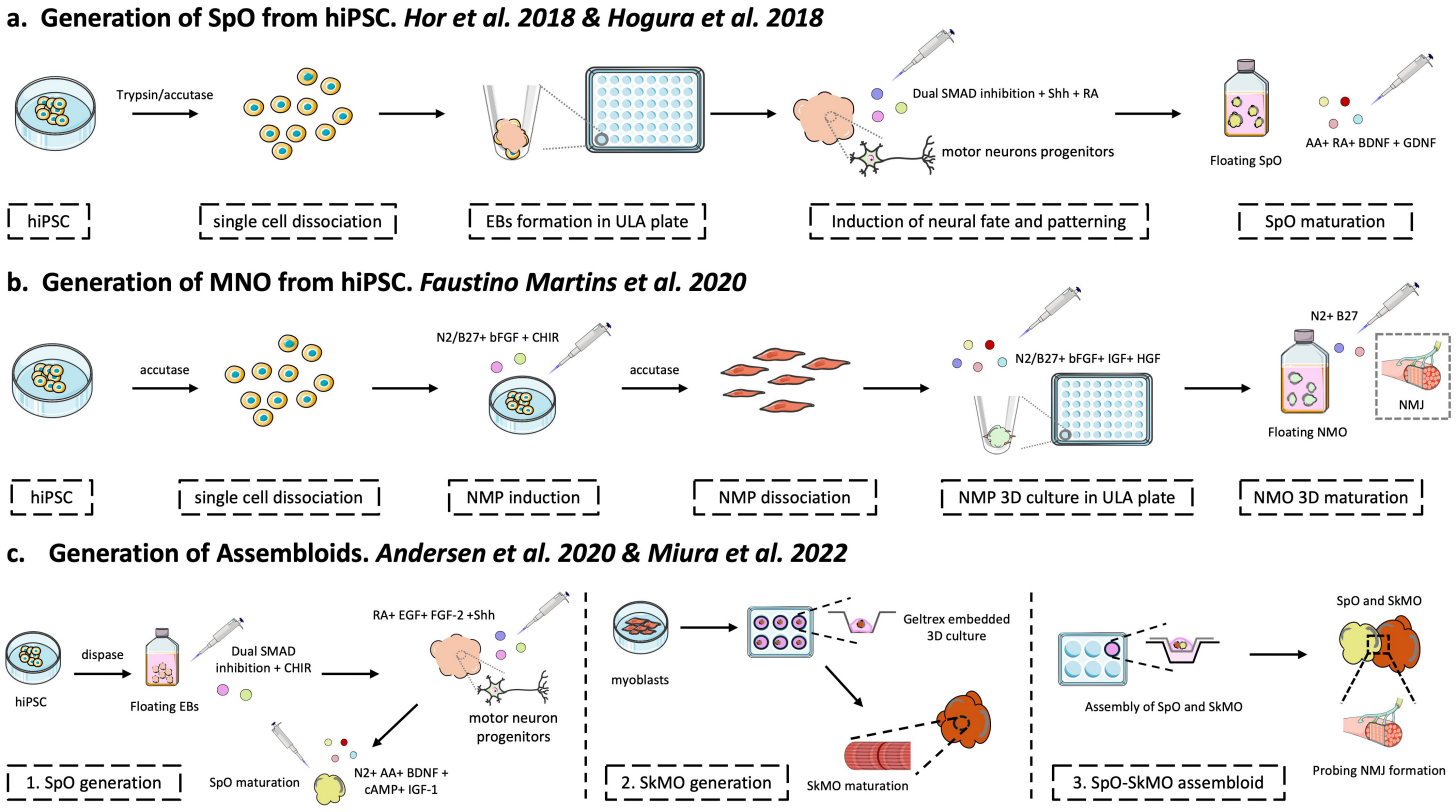
Overview of the protocols for Spinal Organoids (SpO), Neuro-Muscular Organoids (NMO), and Assembloids generation from hiPSC. Here we summarize the most used differentiation strategies for the generation of organoids enriched in motor neurons or a combination of skeletal muscle and motor neurons. Many of the consecutive protocols cited in the review are adaptations to the procedures exemplified in this figure. Changes in media composition, differentiation factors exposure and maturation timing can slightly vary or be mixed from protocol to protocol, as also the technique adopted for Embryoid Bodies (EB) generation and organoids maturation in terms of supports, media and timing. Refer to single publications for detailed information about the differentiation protocol. **(a)** Regarding SpO generation, many publications refers to the works of both Hor and coworkers (70) and Ogura et al. (123), both published in 2018 and displaying many overlapping features. Specifically, hiPSC are enzymatically dissociated with dispase or trypsin to single cells, to be then counted and plated for EB generation in ultra-low attachment (ULA) plates. The EB, that resembles the three germ layers, undergo neural induction through administration of small molecules as LDN-193189 and SB-431542 for Dual SMAD inhibition. Subsequently, neurons ventral and caudal patterning is guided by Retinoic Acid (RA) and Sonic Hedgehog (Shh). The 3D cultures enriched in motor neurons are matured through the administration of RA, ascorbic acid (AA) and two neurotrophic factors as BDNF and GDNF. The SpO maturation is maintained up to day 60 or more. **(b)** MNO generation was firstly described by Faustino Martins and coworkers (75). The procedure is founded on the existence of neuro-muscular progenitor cells that helps in the generation of both skeletal muscle and motor neurons cell population at once. The procedure starts from hiPSC dissociation by accutase, a defined number of cells is seeded and treated with basic FGF and CHIR99021. The latter molecule is an activator of the Wnt/βcatenin pathway and in combination with b-FGF induces the mesoderm commitment. The medium used is supplemented with N2 and B27 in order to sustain neural commitment as well. After neuro-mesodermic induction the resulting neuro-mesodermic progenitors (NMP) are single-cell dissociated to be seeded in ULA plates for tridimensional culture growth. In this phase the 3D cultures are treated with N2, B27, bFGF, IGF and HGF for establishment of both neural and muscular fates. The cited induction lasts for 4 days, later the NMO are matured as floating cultures until day 150 in a medium containing solely N2 and B27. The formation of neuro-muscular junction (NMJ) was demonstrated. **(c)** The father of assembloids is Sergiu Pasca that with his research groups has produced great examples of many different species of organoids' assembloids. We included his work in the figure because, even if it has not been used in ALS or other related pathological contexts, it could represent a valuable alternative to the cited protocols. To this end, we refer to Miura et al. (80) and Andersen et al. (81) works. As the term assembloid suggest, it is generated through the fusion of two separately generated organoids, in this case SpO and skeletal muscle organoid (SkMO). For SpO generation, entire colonies of hiPSC are detached with dispase, these will spontaneously fold into EB. The EB are treated with compounds for Dual SMAD inhibition [see point **(a)**] in combination with CHIR99021, that in this case enhances the formation of neural progenitor cells (NPC) of neural epithelium. The commitment of motor neurons progenitors is achieved through the administration of RA, EGF, FGF-2 and then Shh. Finally, SpO maturation proceeds thanks to neurotrophic factors as BDNF and N2, as also other molecules as IGF-1, AA and cyclic-AMP (cAMP), until the desired endpoint. Simultaneously, SkMO generation starts for myoblasts, that can also be obtained from iPSC (not shown). Myoblasts are dissociated, plated and then grown as tridimensional cultures is extracellular matrix (Geltrex) until they produce the markers of skeletal muscle cells. Once both types of organoids are obtained, they are placed on top of a well insert in proximity towars each other. The fusion of the organoids is spontaneous and generates the assembloids where the motor neurons establish functional connections with muscular fibers to form the NMJ.

Figure 1 describes the three main protocols to derive Spinal Organoids, Neuromuscular organoids and Spinal organoid/Skeletal muscle assembloids (discussed below) with their differences and overlaps. The following slight adaptations to these protocols are not depicted in the figure as the objective extends beyond the systematic analysis of all currently available protocols/adaptations for the generation of the organoids under study.

Comprehensive characterization of spinal cord organoids is now routinely performed through integrated transcriptomic, molecular, and functional analyses. Bulk and single-cell RNA sequencing are widely used to validate regional identity, dorsoventral patterning, and cellular heterogeneity, consistently identifying defined neuronal subtypes (including motor neurons and interneurons), neural progenitors, and, in more mature cultures, astrocytic and oligodendroglial lineages. Spatial specification is further confirmed by immunostaining and *in situ* hybridization for domain-specific markers. Importantly, electrophysiological recordings, calcium imaging, and multielectrode array analyses (MEA) demonstrate the acquisition of intrinsic excitability, synaptic connectivity, and coordinated network activity. Although the relative proportion of individual cell populations varies across protocols and maturation stages, these multi-layered validation approaches robustly support the reproducibility and functional relevance of current spinal cord organoid models (69, 76).

Since the main purpose of the spinal cord is to integrate information between the central nervous system and the periphery (77), it is important to consider that investigating the tissue alone might be insufficient to fully capture its functional role, as alterations in peripheral nerves, muscles, or target organs can critically influence spinal processing and outcomes. To overcome these limits two advanced techniques emerged in the field in recent years: assembloids and microfluidic chips.

Pasca, the first scientist who successfully generated assembloids, describes them “as self-organizing cellular systems resulting from the combination of a type of organoid with another type of organoid” (78, 79). They differ from neuromuscular organoids because they are the product of the fusion between two or more independently specified organoids, already patterned toward a defined identity, meanwhile neuromuscular organoids derived from a shared neuromuscular progenitor that give rise to both derivatives in a coordinated way within the same structure. Dorsal and ventral forebrain organoids, cortical and striatal organoids, or neural organoids combined with non-neural components (Figure 1) can be physically integrated to model inter-regional connectivity or tissue–tissue interactions providing enhanced experimental control (80–83).

On the other hand, conventional organoid or assembloid cultures lack vascularization and controlled perfusion, leading to diffusion-limited transport and heterogeneous microenvironments that complicate the study of specific functional domains, such as synaptic sites or neuromuscular junctions. To address these challenges, microfluidic principles have been applied to organoid models, enabling improved control of the microenvironment and physical compartmentalization, which form the basis of organs-on-chip technologies (84). Controlled flow and pressure within microfluidic platforms help alleviate diffusion limitations of nutrients and morphogens while providing biochemical cues relevant to developmental and physiological processes (85). Furthermore, microfluidic compartmentalization allows synaptic domains and neuron–muscle interaction sites to be spatially organized and independently manipulated, facilitating the study of synaptogenesis and neuromuscular junction formation under defined conditions (86).

### Organoids as platforms to study ALS

The recent advancements in the generation of spinal organoids, characterized by increased complexity and cellular specificity, have led to the development of organoids enriched in ALS-relevant cell types. These systems represent a robust alternative to conventional two-dimensional cultures, as they enable the investigation of disease-associated phenotypes arising from complex multicellular interactions that are insufficiently modeled in simplified *in vitro* approaches. In the following overview, we will summarize the most significant findings in ALS research from 2021 to 2025, that have been achieved using spinal cord organoid models and technologies outlined above.

A critical contribution in this context was provided by Pereira et al. (87), who developed NMOs in which functional neuromuscular junctions formed between motor neurons and skeletal muscle. By comparing organoids derived from ALS patient lines (mutated in *C9orf72* and *FUS)*, genetically edited healthy donors (*SOD1* G85R, *TDP-43* G298S, *PFN1* G118V) and isogenic controls, they observed impairments in NMJ activity, manifested as reduced muscle contractions and altered synaptic marker expression, proving that this model system can recapitulate disease-related characteristics *in vitro*.

Extending these observations beyond the neuromuscular compartment, Szebenyi et al. (88) performed studies on cortical organoids slices derived from ALS patients with *C9orf72* mutations discovering early neural and astrocytes defects compared to the controls, especially in DNA damage and proteostasis. These results suggest a dormant perinatal or presymptomatic cortical vulnerability, a new perspective in the ALS field. Furthermore, the study showed a partially rescued phenotype through GSK2606414 administration. This evidence demonstrates that organoid slices can be used as an efficient drug testing platform. Similar results were also confirmed by Gao et al. (89) in neuromuscular organoids from patient derived cells and their isogenic controls. NMOs displayed the peripheral symptoms of ALS in skeletal muscles and the pathological hallmarks in neurons and astrocytes of the spinal cord. Also, treating organoids with GSK2606414 improved the skeletal muscle defects and reduced the disease hallmarks in a dose-dependent manner.

Studies using patient-derived spinal organoids and isogenic controls (90) identified alteration in the LINC complex, whose function is supporting nuclear positioning, mechanotransduction and chromatin organization (91). These findings suggest that biomechanical dysfunction is an early ALS phenotype conserved in the late stages of the disease, representing a possible biomarker and a target for therapy development.

Genetic perturbation approaches have further reinforced the causal links between ALS-associated genes and organoid phenotypes. For instance, the knock-down of *c9orf72* expression in hiPSCs from healthy donors using a lentiviral shRNA system resulted in a disease-like model, demonstrating that the mutated organoids possess features relevant to ALS and a pro-inflammatory state (92).

Using *c9orf72* hiPSCs derived brain organoids from different donors, Casiraghi et al. (51) studied the behavior of an important pathological hallmark of ALS, TDP-43 mislocalization and aggregates formation. In this study organoids exhibited both TDP-43 cytoplasmic aggregation and nuclear loss-of-function under chronic oxidative stress. Furthermore, they also identified rapamycin as an effective agent in preventing these pathological features, rescuing TDP-43 splicing defects without affecting astrocytes and neural markers.

Beyond Earth-based conditions, organoid models have also been used to explore the effect of microgravity and spaceflight on neuromuscular and cognitive functionality. Sharma et al. (93) investigated the effects of microgravity on motor neurons–astrocyte neurospheroids by comparing healthy samples with disease-like models carrying a TDP-43 knock-in mutation. Their study revealed a significant upregulation of multiple neurodegeneration-associated biomarkers, recapitulating features of various neuromuscular diseases and providing evidence of spaceflight-induced neuropathology. Notably, treatment with NI112 Nanoligomer restored these biomarkers in diseased motor neurons exposed to microgravity to levels comparable to those observed in healthy motor neurons maintained under Earth gravity.

Through an in-depth analysis of brain organoids from presymptomatic and symptomatic patients with a spectrum of *c9orf72* mutations, Van der Geest et al. (94) proved how ALS organoids work as suitable model to recapitulate key aspects of the pathology observed *in vivo*, especially for the early stages. As a matter of fact, many defects found in the organoids from patients at later stages are present already in the early stages. These include the three main molecular hallmarks of c9orf72-related ALS (RNA foci, DPRs, protein haploinsufficiency), impairment in cell populations and distribution together with synaptic detriment.

Technological innovation continues to push the field forward. Shiin et al. (95) successfully developed a biohybrid system combining motor neurons obtained from sALS patient-derived hiPSCs, HUVEC and graphene resulting in improved features in oxygen and nutrients distribution, stem cell growth, neural network development, neurogenesis, and differentiation compared to the standard models.

Furthermore, the NMJ of the biohybrid model presented neuromuscular disease drug effects, demonstrating that the proposed model can be applied to drug screenings and toxicity assessments for diverse neural diseases.

An overview of the evidence, advancements and limitations cited in this section is provided in Table 1.

**Table 1:** Overview of the researches where organoids were used as ALS modelling platforms.

Publication	Organoid type	ALS-relevant genetic background	Key findings	Advantages of the model	Limitations of the model
Pereira et al. (87)	Neuromuscular organoids	Patient-derived iPSCs carrying C9orf72 HRE and FUS mutation. Genome-edited iPSCs from healthy lines: SOD1 G85R, TDP-43 G298S, PFN1 G118V	Patient-derived and mutated organoids show impairments in NMJ activity and altered synaptic markers expression.	i. Physiologically relevant and mutation-specific NMJ modeling ii. Complex multicellular system	i. Reproducibility ii. Lack of vascularization iii. Early-stage phenotype iv. Long term culture
Szebényi et al. (88)	Slices of cortical organoids	Patient-derived iPSCs carrying C9orf72 HRE (~70 and ~800 repeats)	Patient-derived organoids show early neuronal and astrocytic disturbances, including accumulation of dipeptide repeat proteins (DPR), DNA damage, and proteostasis defects; partial phenotypic rescue achieved pharmacologically (GSK2606414).	i. Preserved 3D cortical architecture ii. Long term viability and later stage phenotypes iii. Improved oxygen and nutrient diffusion iv. Experimental accessibility v. Complex multicellular system vi. ALS relevant phenotype	i. Reproducibility ii. Focus on the cortical region, no inclusion of spinal motor neurons and NMJ iii. Slicing artifacts iv. Lack of vascularization v. Early-stage phenotype vi. Long term culture
Gao et al. (89)	Neuromuscular organoids	Patient-derived iPSCs carrying C9orf72 HRE (~800 repeats)	Patient-derived organoids display muscle weakness, denervation, Schwann cell loss, DPR aggregates, and autophagy defects; GSK2606414 improves contraction and reduces aggregates.	i. Physiologically relevant NMJ modeling ii. ALS relevant phenotype iii. Complex multicellular system	i. Reproducibility ii. Early-stage phenotype iii. Lack of vascularization iv. Long term culture
Sirtori et al. (90)	Spinal organoids	Patient-derived iPSCs carrying C9orf72 HRE (~6–8 kb expansions)	Patient-derived organoids show disruption of LINC complex, nuclear envelope integrity and reduced nucleolar size.	i. Highlight of an ALS relevant pathological phenotype that was not present in the 2D model ii. Complex multicellular system	i. Reproducibility ii. Early-stage phenotype iii. Lack of vascularization iv. Long term culture
Guo et al. (92)	Spinal organoids	C9orf72 knockdown in genome-edited healthy iPSCs	C9orf72-knockdown iPSC-derived organoids show elevated expression and secretion of proinflammatory cytokines (IL-6, IL-1β, TNFα, TGFβ)	i. Complex multicellular system ii. ALS relevant phenotype	i. Reproducibility ii. Early-stage phenotype iii. Lack of vascularization iv. Long term culture
Sharma et al. (93)	Motor neuron–astrocyte neurospheroids	TDP-43 overexpression in genome-edited healthy iPSCs	TDP-43 overexpressing organoids show increased TDP-43 and KLK6 levels following spaceflight exposure; treatment with nanoligomers partially reduced neurodegenerative markers, though effects were not statistically significant.	i. Complex multicellular system ii. ALS relevant phenotype	i. Reproducibility ii. Early-stage phenotype iii. Lack of vascularization iv. Long term culture v. Limited translational potential
Van der Geest et al. (94)	Brain organoids	Multiple patient-derived iPSC lines carrying C9orf72 HRE (~666–1,175 repeats)	Patient-derived organoids show C9ORF72 haploinsufficiency, RNA foci, DPRs; altered organoid; reduced deep-layer cortical neurons and disorganized radial glia; synaptic deficits in excitatory neurons; phenotypes variable among presymptomatic carriers.	i. Complex multicellular system ii. Pre-symptomatic phenotype	i. Reproducibility ii. Early-stage phenotype iii. Lack of vascularization iv. Long term culture
Casiraghi et al. (51)	Brain organoids	Patient-derived iPSCs carrying C9orf72 HRE (3 lines with different repeat expansions)	Patient-derived organoids show TDP-43 loss-of-function, P62 accumulation, and splicing defects in downstream target genes upon chronic oxidative stress; rapamycin treatment rescues TDP-43 splicing defects, without affecting neuronal or astrocytic markers.	i. Complementary analysis of 2D and 3D ii. Complex multicellular system iii. Pre-symptomatic phenotype	i. Reproducibility ii. Lack of vascularization iii. Long term culture
Shin et al. (95)	Biohybrid spheroid composed of graphene/HUVEC/neural cell combined with skeletal muscle bundles	Patient-derived iPSCs from sporadic ALS (sALS) patients	Patient-derived spheroids show TDP-43 accumulation and reduced neurofilament expression, leading to impaired neurite outgrowth and muscle contraction; treatment with bosutinib restores NMJ function, enhances neurite growth, and reduces TDP-43 levels.	i. Presence of a vascular component ii. Enhanced neural network activity iii. Platform suitable for drug testing iv. Limited cell type diversity	i. Complex biofabrication ii. Lower complexity of spheroid compared to organoids

To date, SpO or NMO have been used for low throughput drug screening in both SMA and ALS context (88, 89, 96). Due to the efficient recapitulation of disease phenotype, organoids enable the testing of almost the same parameters normally probed in traditional monolayer cultures, serving as a more informative platform for drug screening and giving very promising results (97–99).

The possibility of automation, to standardize organoids dimensions and growth, opens the way toward high-throughput drug testing, as demonstrated in other studies on midbrain organoids and Alzheimer's Disease (100, 101). The incredibly fast proceedings in organoid research together with organoids' human genetic background could help in overcoming the lack of clinical translational power held by mice models. Nevertheless, we are still quite far from replacing murine models completely with organoids, especially for pharmacodynamics and pharmacokinetics studies.

## Clinical applications and limitations

The broader organoid field has progressively transitioned from purely developmental modeling toward translational and clinically oriented applications, supporting the expectation that similar trajectories may emerge for neurodegenerative disorders such as ALS.

Patient-derived intestinal organoids have been used to functionally assess CFTR activity and predict individual responses to CFTR modulators in cystic fibrosis, establishing a direct correlation between *ex vivo* drug testing and clinical benefit (102). In oncology, tumor-derived organoids from colorectal, pancreatic, and gastric cancers are employed to evaluate drug sensitivity and generate living biobanks that inform personalized treatment strategies (103–105). Organoid systems are also incorporated into preclinical drug development and toxicity screening, including liver, cardiac, and kidney models to assess efficacy and off-target effects in a human tissue context (106, 107). Moreover, organoid platforms have been utilized to model host–pathogen interactions and complex multicellular responses relevant to therapeutic development (108). Although spinal and neuromuscular organoids for ALS have not yet reached comparable levels of clinical implementation, these precedents demonstrate the feasibility of integrating organoid-based systems into precision medicine and drug development workflows, supporting their potential future role within the ALS translational landscape.

Overall, organoid models offer substantial advantages over traditional 2D cultures and animal models, providing a three-dimensional, human-specific context that better recapitulates aspects of tissue architecture, cellular heterogeneity, and disease-relevant physiology (109). They allow for patient-derived modeling, enabling the study of individual genetic backgrounds and early pathological events that are often difficult to capture *in vivo* (110). These systems have facilitated major advances in understanding motor neuron degeneration, synaptic dysfunction, and NMJ pathology in ALS.

However, several limitations must be considered. Many ALS-related studies using organoids have focused on narrow aspects of ALS pathology, such as NMJ functionality or alterations in the nuclear envelope (87, 89, 90), often relying on genetically engineered lines rather than fully patient-derived models which may limit translational relevance (92, 93). This aspect could be enhanced by leveraging patient-derived cell samples from established biobanks, such as EBiSC (https://ebisc.org/) and the Cedars-Sinai Biomanufacturing Center (https://csbiomfg.com/), where many cell lines are available together with their disease-corrected isogenic counterparts. To achieve a stronger translational impact, researchers should foster closer collaborations with clinicians to facilitate access to patient biopsies. In addition, for laboratories with limited expertise in molecular biology, specialized service providers offer reprogramming and gene-editing solutions, including CRISPR–Cas9 technologies. Although these strategies may not immediately overcome current translational limitations, they represent essential steps toward improving long-term translational outcomes.

Furthermore, the reproducibility of neural and neuromuscular organoids remains a significant challenge, with variability arising from differences in iPSC lines, differentiation protocols, and culture conditions (111). This variability can complicate the comparison of results across studies and the interpretation of subtle phenotypes. Nonetheless, efforts have been made to improve organoid reproducibility and reduce variability through the implementation of automated procedures (100), which could substantially decrease batch-to-batch differences. However, a major challenge still lies in the protocol adjustments that are often based on the specific expertise and instrumentation available in each research group.

Other general limitations include limited rigor, poor model validation, and a lack of unbiased analysis or hypothesis validation (112). The reliance on specific markers or antibodies to identify cell types of interest may introduce bias, potentially overlooking inappropriate cell populations. Additionally, the lack of unbiased, high-throughput characterization methods can mask heterogeneity within organoids, reducing the reliability and generalizability of findings.

Nevertheless, in the last years emerged the need to perform high content analysis (HCA) for unbiased characterization and validation. These analysis include single cell transcriptomics (scRNAseq) and other -omics such as proteomics, metabolomics and lipidomics, but also high content imaging (113, 114). Unfortunately, these technologies are not accessible to all research groups and add up to the already substantial costs associated with organoid culture and maintenance.

In many current models, motor neurons and myotubes are co-cultured or co-developed within the same 3D environment, creating unnaturally proximity between neuronal cell bodies and muscle fibers. This arrangement may affect NMJ formation, resulting in reduced acetylcholine receptor clustering, impaired colocalization of pre- and postsynaptic markers, and altered neurotrophic signaling along axons (115, 116). Consequently, these systems may fail to capture key aspects of ALS pathology, such as distal axonopathy, transport deficits, and progressive NMJ denervation preceding somatic degeneration. These limitations could be addressed through bioengineering approaches, such as 3D bioprinting and microfluidic systems, which aim to recapitulate vascularization while ensuring functional compartmentalization of organoids in a quasi-physiological context (117–120).

In general terms, it is widely acknowledged that current organoid systems more faithfully recapitulate early human neurodevelopment than late-stage neurodegeneration, given their relative immaturity and limited aging signatures. However, this apparent limitation may also represent a conceptual opportunity as increasing evidence suggests that many neurodegenerative disorders, including ALS, Parkinson's disease, and Alzheimer's disease, may originate from subtle developmental alterations, early network dysfunction, or intrinsic cellular vulnerabilities that precede overt neuronal loss (121, 122). In this context organoids provide a unique platform to investigate early pathogenic cascades, including altered neuronal specification, proteostasis imbalance, stress response dysregulation, and circuit-level hyperexcitability, all events that cannot be recapitulated in *post mortem* tissues.

Ultimately, the utility of organoid models in ALS research will depend on the field's ability to balance biological complexity with experimental rigor. Continued efforts toward reproducibility, unbiased validation, and anatomical fidelity will determine whether these systems can move from exploratory tools to reliable frameworks for understanding and treating motor neuron degeneration.

## Concluding remarks

In conclusion, iPSC-derived spinal and neuromuscular organoids represent a transformative advance in ALS research by faithfully recapitulating critical aspects of disease pathology, including motor neuron degeneration, TDP-43 dysfunction, neuromuscular junction impairment, and non–cell-autonomous contributions from glial cells. These models uniquely bridge the gap between traditional 2D cultures, animal models, and the complex human nervous system, offering an unprecedented opportunity to study ALS pathogenesis in a physiologically relevant, patient-specific context.

Unlocking the full translational potential of spinal and neuromuscular organoid in ALS studies will depend on overcoming critical technical and methodological challenges. Priority areas include standardization of differentiation and quality control protocols, enhancement of organoid maturation and complexity through vascular, immune, and long-range neural integration, and development of scalable culture systems to support high-throughput screening. Multi-omics profiling and collaborative data sharing will further accelerate biomarker discovery and functional validation. By addressing these challenges and consolidating methodological standards, spinal and neuromuscular organoids can become robust, clinically relevant platforms, capable of bridging mechanistic research and therapeutic development, ultimately advancing precision medicine approaches for ALS.
